# Groin abscess outside the hernia sac secondary to perforated appendicitis in an older adult: a case report

**DOI:** 10.1093/jscr/rjag466

**Published:** 2026-06-15

**Authors:** Nobuyuki Okamoto, Takaoki Furukawa, Toshinori Hirano, Ichiro Nagamine, Sunao Otagaki, Shinya Takahashi

**Affiliations:** Department of Surgery, Hiroshima Kyoritsu Hospital, 2-20-20 Nakasu, Asaminami-Ku, Hiroshima, Hiroshima 731-0121, Japan; Department of Surgery, Graduate School of Biomedical and Health Sciences, Hiroshima University, 1-2-3 Kasumi, Minami-ku, Hiroshima, Hiroshima 734-8551, Japan; Department of Surgery, Hiroshima Kyoritsu Hospital, 2-20-20 Nakasu, Asaminami-Ku, Hiroshima, Hiroshima 731-0121, Japan; Department of Surgery, Hiroshima Kyoritsu Hospital, 2-20-20 Nakasu, Asaminami-Ku, Hiroshima, Hiroshima 731-0121, Japan; Department of Surgery, Hiroshima Kyoritsu Hospital, 2-20-20 Nakasu, Asaminami-Ku, Hiroshima, Hiroshima 731-0121, Japan; Department of Surgery, Hiroshima Kyoritsu Hospital, 2-20-20 Nakasu, Asaminami-Ku, Hiroshima, Hiroshima 731-0121, Japan; Department of Surgery, Graduate School of Biomedical and Health Sciences, Hiroshima University, 1-2-3 Kasumi, Minami-ku, Hiroshima, Hiroshima 734-8551, Japan

**Keywords:** right groin abscess, acute appendicitis, groin swelling

## Abstract

Groin abscesses secondary to acute appendicitis are rare, particularly when inflammation spreads retroperitoneally and the abscess forms outside the hernia sac rather than within such as occurs in Amyand’s hernia. An 84-year-old woman with advanced breast cancer and a known inguinal hernia developed a progressively enlarging right groin mass 4 days after mastectomy. Computed tomography revealed a fluid collection, and needle aspiration confirmed an abscess. Conservative management led to temporary improvement; however, a recurrent abscess near the cecum appeared 31 days later. Surgical intervention revealed a partially perforated appendix, indicating retroperitoneal extension of appendiceal inflammation as the cause of the groin abscess, and appendectomy was performed. The patient recovered without recurrence at the three-month follow-up. Acute groin swelling in patients with inguinal hernia should prompt suspicion for appendicitis-related abscess, even without typical abdominal symptoms. Early imaging is essential, particularly in older adults with atypical presentations.

## Introduction

Acute appendicitis occasionally results in localized inflammatory masses or intra-abdominal abscesses; however, extension of appendiceal inflammation toward the groin is exceedingly rare [1, 2]. Most of the groin infections associated with appendicitis occur in the context of an Amyand’s hernia, in which the appendix is incarcerated within the hernia sac [3, 4]. In contrast, retroperitoneal spread of appendiceal inflammation leading to a groin abscess outside the hernia sac represents an uncommon and diagnostically challenging entity. We report a rare case of a groin abscess caused by perforated appendicitis in an older adult, in whom the diagnosis was delayed owing to atypical clinical presentations.

## Case report

**Figure 1:**
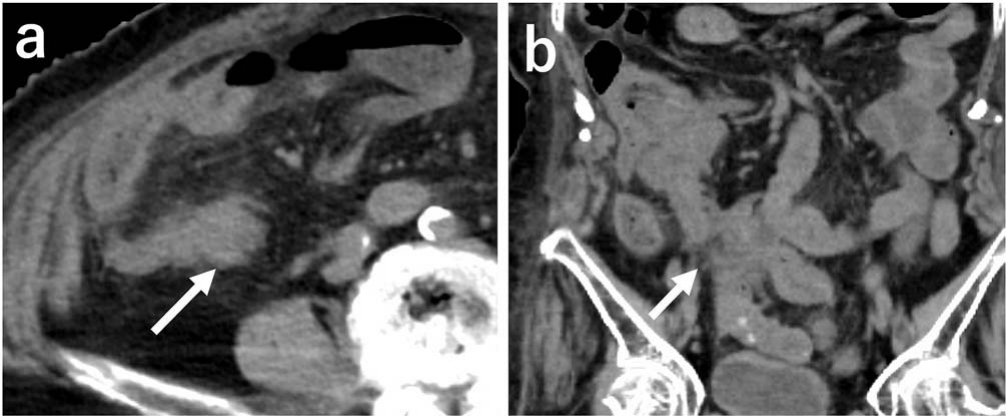
CT 28 days before mastectomy. (a–b) Mild fluid collection can be seen around the appendix.

An 84-year-old woman with a history of dementia necessitating 24-hour care for activities of daily living, and cerebral infarction, multiple lumbar compression fractures, a right femoral neck fracture, and an untreated right inguinal hernia presented to our hospital with a left-sided breast lump. The patient was diagnosed with left intramammary mucinous carcinoma, and a mastectomy with sentinel lymph node biopsy was performed. Twenty-eight days before the mastectomy, blood tests had revealed a white blood cell (WBC) count of 11 300/μL. Preoperative whole-body computed tomography (CT) showed mild fluid collection around the appendix (Fig. 1a and b). However, this finding was not initially considered clinically significant, and the mastectomy was performed as scheduled.

**Figure 2:**
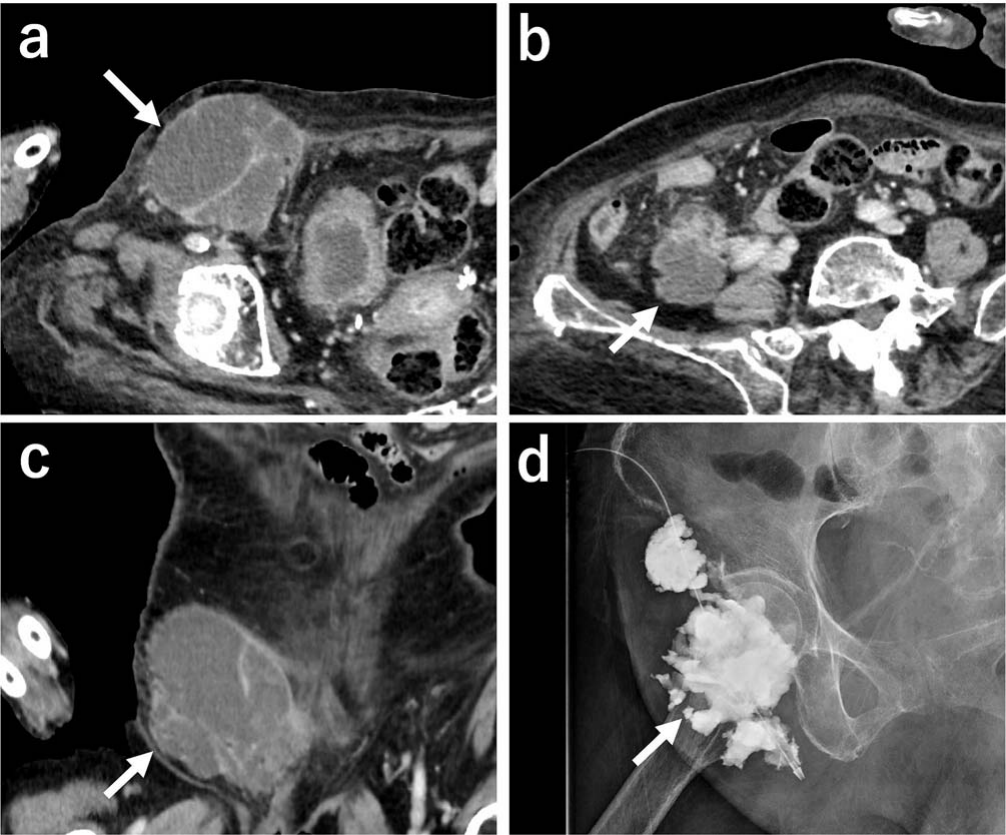
Postoperative imaging. (a–c) Abdominal CT 4 days after mastectomy. The fluid collection can be seen in the right groin area and around the cecum. The collection had formed outside the hernia sac at the groin. (d) Contrast imaging of the abscess. No communication between the abscess and the intestinal tract is observed.

On postoperative day four, a progressively growing mass was observed in the right groin. Abdominal CT revealed fluid collection in the right groin and around the cecum (Fig. 2a–c). The fluid collection appeared isolated, with no obvious continuity with surrounding structures. Aspiration from the area of fluid collection revealed purulent fluid. Contrast-enhanced imaging of the abscess revealed no intestinal communication (Fig. 2d). The patient had no pain around the cecum, and the symptoms did not suggest appendicitis. A mild fever and the elevated WBC count of 12 860/μL were considered as postoperative effects and/or as indicative of a groin infection. A drainage tube was inserted into the groin area and conservative management with antibiotics was initiated. The swelling subsided and laboratory findings gradually improved. The patient was discharged 19 days later.

**Figure 3:**
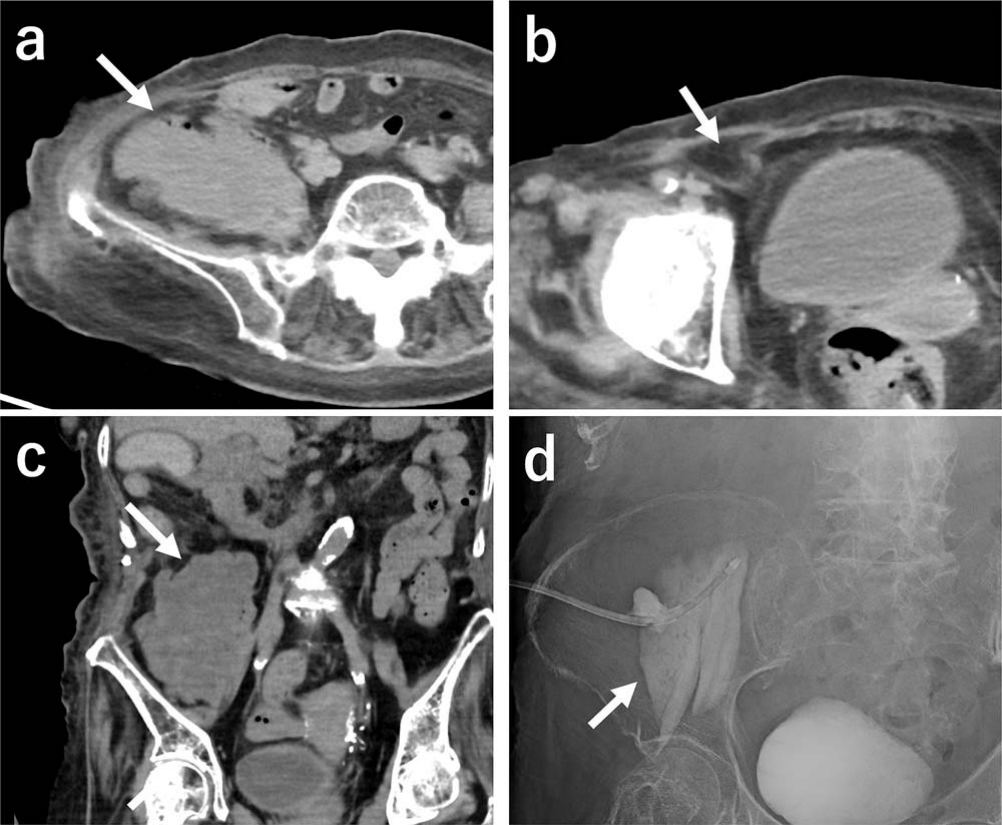
Imaging after readmission. (a–c) Abdominal CT. Fluid collection is present around the cecum but not in the groin area. (d) Contrast imaging of the abscess. No communication is evident between the abscess and the intestinal tract.

Thirty-one days later, the patient returned with decreased oral intake over several days. Abdominal CT revealed fluid collection around the cecum (Fig. 3a–c). Contrast-enhanced imaging revealed no intestinal communication with the area of fluid collection (Fig. 3d). Conservative treatment was attempted; however, after two days, intestinal fluid began to drain from the area. Despite continued conservative treatment, the patient’s condition did not improve, and surgical intervention was deemed necessary. A laparotomy was performed through a midline abdominal incision. A perforation was found in the appendix, which was thought to have contributed to the abdominal and groin abscesses. Appendectomy was performed, and a drainage tube was placed in the retroperitoneal cavity.

The postoperative recovery was uneventful, and the patient was discharged on postoperative Day 17. As of the three-months postoperative follow-up, no abscess recurrence or inguinal hernia-related symptoms have been observed.

## Discussion

Groin abscesses secondary to acute appendicitis are rare, and most previously reported cases have involved Amyand’s hernia, in which the appendix itself is incarcerated within the hernia sac and becomes inflamed or perforated [3, 4]. In contrast, the present case represents a distinct pathophysiological mechanism: acute appendicitis developed intra-abdominally, and the subsequent inflammation spread retroperitoneally toward the groin, ultimately forming an abscess adjacent to, but not within, the hernia sac. This pattern of extension differs fundamentally from Amyand’s hernia and should be recognized as a separate clinical entity.

The diagnostic challenge in this case was compounded by the patient’s advanced age and atypical presentation. Prior to the onset of the groin mass, the patient exhibited neither high fever nor localized right lower abdominal pain, which are typical symptoms of appendicitis. Such muted symptoms are common in older adults and can easily delay diagnosis. A retrospective review of the patient’s course of treatment and diagnostic tests before the mastectomy revealed that the preoperative blood tests and CT findings suggested the possibility of appendicitis. This highlights the importance of carefully interpreting incidental findings in older adult patients undergoing surgery for other conditions.

CT imaging played a crucial role in clarifying the disease process. The abscess was located outside the hernia sac, supporting the hypothesis that the inflammation tracked along the prerenal fascia and lateral conus fascia, as previously described in retroperitoneal-origin groin abscesses [5]. The presence of an inguinal hernia likely provided a potential space that facilitated fluid accumulation and abscess formation. Recognizing this anatomical pathway is essential for differentiating this condition from Amyand’s hernia, De Garengeot hernia [6], and other causes of groin swelling [5, 7, 8].

Management of groin abscesses requires careful consideration of infection risk. Surgical strategies generally fall into two categories: one- and two-stage procedures. One-stage surgery, such as iliopubic tract repair or the McVay method, involves tissue- to- tissue repair. The two-stage surgery consists of appendectomy and abscess drainage, followed by delayed tension-free hernia repair using a mesh. Because tissue-to-tissue repair is associated with higher recurrence rates, delayed tension-free repair is generally preferred when infection can be controlled. However, in frail older adults with multiple comorbidities, forging hernia repair entirely may be the most reasonable and feasible option, as adhesions following abscess formation may reduce the hernia cavity. This case underscores the need for individualized surgical planning based on anatomical findings, infection severity, and patient condition.
